# Relationship between haemagglutination inhibition titre and immunity to influenza in ferrets

**DOI:** 10.1016/j.vaccine.2015.08.065

**Published:** 2015-10-05

**Authors:** Paul S. Wikramaratna, Andrew Rambaut

**Affiliations:** a Institute of Evolutionary Biology, University of Edinburgh, UK; b Fogarty International Center, National Institute of Health, Bethesda, MD, USA; c Centre for Immunology, Infection and Evolution, University of Edinburgh, Edinburgh EH9 3FL, UK

**Keywords:** Haemagglutination inhibition assay, Influenza, Ferrets, Antigenic evolution

## Abstract

Our understanding of the antigenic evolution of the human influenza virus is chiefly derived from experiments in which serum from influenza infected ferrets is tested against panels of virus isolates in the haemagglutination inhibition (HI) assay. The interpretation of these results has been much aided by the development of antigenic mapping techniques, which suppose that the antigenic distance between two different influenza viruses is directly proportional to their fold-difference in titre in this assay. Yet, antigenic distance is not necessarily the same as cross-protection, and high levels of protection have been observed in humans against strains to which they have low HI titres. However, no study has previously addressed the relationship between HI titre and cross-protection in ferrets: the standard animal model. This study fills this gap by analysing published data where pre-challenge HI titres are available for individual ferrets, and post-challenge outcomes have been recorded. Ultimately, this work confirms that it is the absolute, rather than relative, HI titre that determines the extent of immunity and that there is a threshold HI titre beyond which ferrets are completely protected from infection. Nevertheless, this titre is much higher in ferrets than has been suggested for humans. Further, we are consequently able to show that using distance between strains within an antigenic map to predict cross-protection between influenza viruses can be misleading.

## 1 Introduction

Antigenic evolution is driven by selection for onward transmission: pathogens that can vary their antigenic properties may create opportunities to re-infect immunologically experienced hosts. Influenza is one such pathogen. Many mutants are generated as it replicates, enough of which have sufficiently altered antigenic properties to drive recurrent influenza epidemics in humans. The burden of these epidemics is such that we take care to vaccinate those most at risk from infection, but the pace of antigenic evolution means that we must expend exhaustive and expensive efforts to keep the vaccine relevant.

Attempting to understand past, and hence perhaps predict future, trajectories of influenza within antigenic space is of interest, both theoretically and practically. The primary (but by no means only) tool for this is the haemagglutination inhibition (HI) assay, a two-fold dilution assay in which the ability of serum to prevent the agglutination of red blood cells by a particular influenza virus is measured. HI titres are generally reported as the reciprocal of the highest dilution at which agglutination was prevented, with increasing dilutions indicating higher degrees of antigen binding. The assay can be criticised because it is often poorly reproducible between different laboratories [1–3], can only measure the ability of antibodies to prevent the binding of influenza to cells that the virus does not target *in vivo*, and assumes that ferrets and humans develop identical cross-reactive immune responses when challenged with the virus (for more detail see [4–6]). Nevertheless, it remains the gold standard within influenza research. Its results are of critical importance in the process of annual vaccine strain selection: does the serological response of a ferret (the standard animal model for human influenza) vaccinated with strain X suggest that they would be protected against the apparently emergent strain Y? If not, then the vaccine strain may need to be updated.

A seminal paper in this area introduced the concept of antigenic cartography [7]. The authors based their work on the reasoning that if a ferret that has been previously infected by strain X has an HI titre of P against X, and a titre of Q against another strain, Y, then the difference between P and Q tells us something about the proximity of strains X and Y within antigenic space. A two-fold difference in titre is taken to correspond to 1 unit of distance within this space.

The method employs multi-dimensional scaling to represent the past antigenic evolution of the virus within a two-dimensional space and it is now exceedingly rare for antigenic data to be presented without an accompanying antigenic map. The original application of these methods to 35 years of human A/H3N2 evolution illustrated that the antigenic evolution of human influenza is more punctuated than continuous: viruses cluster together in antigenic space for a number of years before suddenly jumping to a new point and the average distance between the identified antigenic clusters of H3N2 was 4.5 units. Subsequent applications to A/H1N1 and influenza B have revealed broadly similar patterns, although both appear to ‘jump’ somewhat less energetically [8].

There is, however, a potential difference between antigenicity (literally: the ability to bind to an antigen) and immunogenicity, or cross-protection. Indeed, a previous analysis of pre-challenge HI titre and its effect on the probability of a serological response in humans has suggested that a titre against a challenge strain as low as 17 may provide a 50% chance of protection against infection [9,10]. Not only is this titre notably below the normal threshold for detection in the assay (a titre of 40), but the results also suggest that any titre in excess of 200 is almost completely protective. Thus, for example, if antiserum raised against strain X has a homologous HI titre of 5120, but HI titres of only 1280 against strain Y and 80 against strain Z, then strains Y and Z are said to be 2 and 6 units of antigenic distance from X. Contrastingly, in terms of cross-protection, the above results suggest not only that infection with strain X provides complete protection against strain Y, but also that infection with X provides significant protection against strain Z.

Nevertheless, this analysis considers protection from infection in natural human infection, as opposed to experimental infection in ferrets, which is the actual source of almost all antigenic data and inference. Whether the relationship between HI titre and cross-protection is the same in ferrets as it is in humans is therefore important. Although this question has not been previously considered, relevant data has been presented in a number of different papers and is analysed together here. In particular, we focus on the results of a series of experiments in ferrets in the 1970s using the 1934 H1N1 and 1968 H3N2 viruses.

An advantage of the present study is that previous work on cross-protection in humans [9,10] could only try to infer if an individual had been infected by whether they had a four-fold or greater rise in HI titre over the course of the flu season (this is the definition of seroconversion [11], but see [12]). Yet cross-protection between strains must be related to the reduction in transmission of one following prior exposure to the other: something about which a serological response may itself tell us little. Fortunately, data on both the change in HI titre against a particular strain upon challenge and the viral titre in nasal wash taken after challenge has been presented for individual ferrets [13–19].

We therefore use this data to estimate the relationship between HI titre and (i) prevention of a serological response and (ii) reduction of virus production (and hence transmission) in ferrets. We then compare our results to those of [9] and consider the consequences for our understanding of the antigenic evolution of influenza.

## 2 Materials and methods

Using the keywords “immunity”, “influenza” and “ferret” we identified papers in PubMed that might contain appropriate data. We then included data from studies where ferrets had pre challenge HI titres against a specific strain recorded, with post challenge measurements of HI titre and/or the amount of virus produced in nasal wash but excluded those ferrets that had been previously vaccinated or challenged with a strain that was HA-mismatched but NA-matched with the subsequent challenge strain (e.g. previously challenged with H1N2, measurements provided for subsequent challenge with H3N2). This resulted in 162 observations from 7 papers [13–19] (Supplementary Table S1).

### 2.1 Probability of a serological response

Typically, a four-fold rise in HI titre following challenge is taken as a positive serological response and is indicative of a successful infection; this was the measure used in the studies analysed by Coudeville et al. [9]. Following their lead, we start with a baseline probability (*P*) that an individual naïve ferret will exhibit a serological response when challenged with an influenza strain. To estimate the reduction in this probability in the presence of HI antibodies, this baseline probability is combined with a function (0 ≤ *ρ*(*T*_*j*_, *η*) ≤ 1, where *T*_*j*_ is the HI titre and *η* is the associated vector of parameters) such that the probability of a serological response in a ferret upon exposure is

$$R\left(P,T_{j},\eta \right)=P*(1-\rho (Tj,\eta ))$$

In line with Coudeville et al., we will specify the functional form associated with *ρ*(*T*_*j*_, *η*) as a two parameter inverse logit function (*η* = {*χ*, *ϕ*}) applied to log-transformed HI titre values:

$$\rho \left(T_{j},\eta \right)=\frac{e^{\phi (\log_{2}\left(T_{j}\right)-\chi )}}{1+e^{\phi (\log_{2}\left(T_{j}\right)-\chi )}}=1-\frac{1}{1+e^{\phi (\log_{2}\left(T_{j}\right)-\chi )}}$$

and so

$$R(P,T_{j},\chi ,\phi )=\frac{P}{1+e^{\phi (\log_{2}\left(T_{j}\right)-\chi )}}$$

Coudeville et al. point out that *ϕ* corresponds to the titre that halves the amount of virus produced (it is a location parameter for the curve) whilst *χ* determines its steepness [9]. The main reason for choosing a two-parameter inverse logit function, as opposed to a different smooth increasing function, is that it leads to a straightforward method of constructing confidence intervals for the probability of protection. It is also the standard ‘link function’ for binary data so represents a natural choice here. For more information see [20].

### 2.2 Reduction in virus production in undiluted nasal wash

In these papers, the amount of virus produced in undiluted nasal wash is reported 3 days after challenge as 10^X^ EID50/ml (50% egg infectious dose per ml). In this paper we will model the effect of HI titres on reducing the size of this exponent.

The model estimates the amount of virus, *V*, which an individual ferret will produce when inoculated with influenza. In the absence of HI antibodies, the average amount, *μ*, corresponds to a baseline, γlog_10_ EID50/ml. This value may be reduced in the presence of HI antibodies however, and is therefore combined with a function describing the effect of HI titre (0 ≤ *π*(*T*_*j*_, *θ*) ≤ 1, where *T*_*j*_ is the HI titre and *θ* is the associated vector of parameters). Thus, the average amount of amount of virus produced by an exposed ferret is

$$\mu (\gamma ,T_{j},\theta )=\gamma (1-\pi (T_{j},\theta ))$$

As above, we will specify the functional form associated with *π*(*T*_*j*_, *θ*) as a two parameter inverse logit function (*θ* = {*α*, *β*}) applied to log-transformed HI titre values:

$$\pi \left(T_{j},\alpha ,\beta \right)=\frac{e^{\beta (\log_{2}\left(T_{j}\right)-\alpha )}}{1+e^{\beta (\log_{2}\left(T_{j}\right)-\alpha )}}=1-\frac{1}{1+e^{\beta (\log_{2}\left(T_{j}\right)-\alpha )}}$$

As before, *α* corresponds to the titre that halves the amount of virus produced (it is a location parameter for the curve) whilst *β* determines its steepness.

Finally, we anticipate that not all ferrets will respond identically: the actual amount of virus produced will vary around some mean. In particular, following inspection of the data (see Supplementary Figure S1), we suppose that it is lognormally distributed around *μ* with standard deviation *σ* (which is to be estimated). And so, the amount of virus produced by a ferret upon exposure is

$$V\left(\gamma ,T_{j},\alpha ,\beta ,\sigma \right)∼N\left(\frac{\gamma }{1+e^{\beta (\log_{2}\left(T_{j}\right)-\alpha )}},\sigma \right)$$

### 2.3 Censored data

An important consideration is that reported HI titres represent interval censored data. If a titre is reported as ‘X’ then the true titre in fact lies in the interval [X, 2X), whereas if it is reported as ‘<Y’ then it lies on the interval [1,Y). We therefore treat the HI titres as latent variables that exist somewhere in the appropriate interval (as opposed to assigning them an arbitrary value somewhere within this interval). The titre is reported as ‘>5120′ for one observation. Strictly speaking this means that the true titre lies on the interval (5120, ∞), but because this titre is already very large we choose to model it as a latent variable on (5120, 10.240) for practical reasons.

There also appears to be a detection limit for the amount of virus produced in nasal wash, which is recorded as 0.7log_10_ EID50/ml in one study [21] (NB No ferrets in this study were actually included in the analysis here because HI titres against the actual challenge virus were not recorded). In many instances, viral titres are reported as ‘0′ rather than as ‘<Y’ but we believe that the former is shorthand for the latter. For all viral titres reported as 0, we have therefore chosen to model the titre as a latent variable that exists somewhere in [0,0.7).

### 2.4 Parameter estimation

Markov Chain Monte Carlo (MCMC) methods were implemented on the models specified above, using the software package ‘rjags’ [22].

Posterior summary statistics were based on 3 Markov chains of 20,000 lengths after a burn-in of 40,000 iterations. Convergence was assessed using Gelman–Rubin statistics [23].

For each of the unknown parameters specifying the shape of a curve (*α*, *β*, *γ*, *χ*, *ϕ*) we chose a standard diffuse prior ∼ *Γ*(0.0001, 0.0001) (so that these parameters could take on any value in (0, *∞*)), but for P chose a prior ∼ *U*(0,1).

## 3 Results

Supplementary Figures S2 and S3 and the Gelman–Rubin statistics suggest that the Markov chains and burn-in period are sufficiently long for the posterior statistics to be meaningful.

**Fig. 1:**
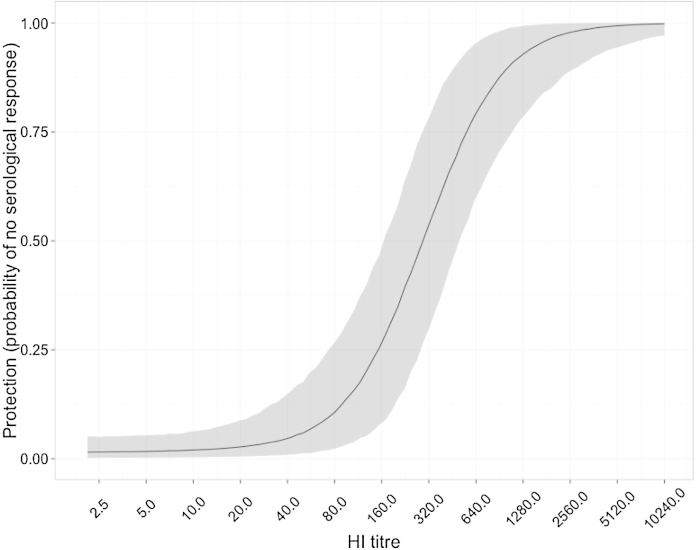
Estimated effect of pre-existing HI titre on probability of serological response. The solid black line shows the posterior median value, with the 95% credible interval shaded in grey.

**Fig. 2:**
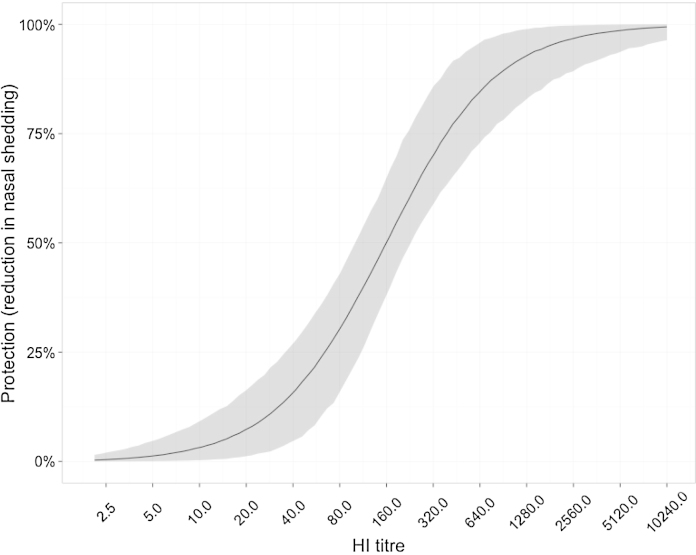
Estimated effect of pre-existing HI titre on extent of nasal shedding. This is the reduction in the average amount of nasal shedding predicted by a particular HI titre, as compared to the expected titre when the HI titre is undetectable. The solid black line shows the posterior median value, with the 95% credible interval shaded in grey.

Parameter estimates are presented in Tables 1 and 2; we estimate the 50% protective titre against seroconversion as 300 (95% CI 170–508) and the titre that leads to a 50% drop in log_10_ virus titre in nasal wash as 159 (95% CI 98–228). The respective curves are shown in Figs. 1 and 2. Notably, there is a threshold HI titre at which undetectable amounts of virus are produced in nasal wash (for titres in excess of 640 the viral titre drops below 10^0.7^; see Fig. 2). Further increases in HI titre beyond this threshold are therefore irrelevant with respect to these measures.

**Fig. 3:**
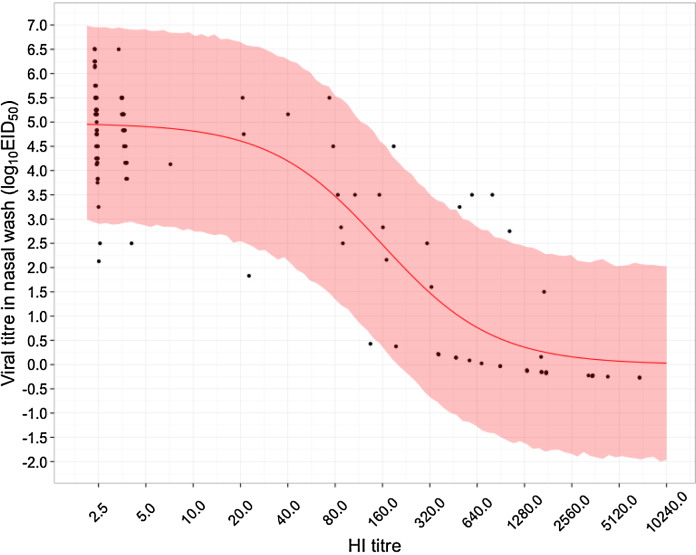
Predicted viral titre based on pre-existing HI titre. The solid red line shows the median level of protection predicted by pre-existing HI titre, while the shaded region shows the 95% CI. The data on which the analysis is based are represented by black circles. There is some uncertainty in the exact HI measurement for all ferrets as the pre-challenge HI titre of each ferret is interval censored data; what is shown here is the median posterior estimate of the true HI titre. Similarly, for ferrets whose viral titre was described as undetectable (“<5” or “<10”), shown here is the median posterior estimate of the true viral titre. In other words, the black dots represent a best guess of the true titre for each ferret, given the prior knowledge of the interval on which their HI titre lies, the observed viral titre and the inferred relationship between pre-existing HI titre and viral titre.

Unsurprisingly, higher HI titres result in both a lower probability of a serological response and a lower volume of virus shedding. Our results suggest, however, that the effect on virus shedding is more potent. This makes intuitive sense: seroconversion signals that the pre-existing immune response was insufficient to clear the infection and so might be triggered only by large amounts of virus production. Nevertheless, the credible intervals for both curves overlap and so a definitive statement is impossible. One reason for the width of these credible intervals is that data in the apparently critical region is comparatively sparse: there are relatively few ferrets with intermediate pre-challenge HI titres (Fig. 3).

The parameter *σ* is necessary to capture the full extent of variability in the amount of virus produced in nasal wash by ferrets (Fig. 3), which presumably reflects experimental noise, such as the varying resistance of individual ferrets to infection. Coupled with the general lack of ferrets whose HI titres are in the critical region, this indicates a need for more experiments to be carried out in immunologically experienced ferrets to better characterise these relationships.

**Fig. 4:**
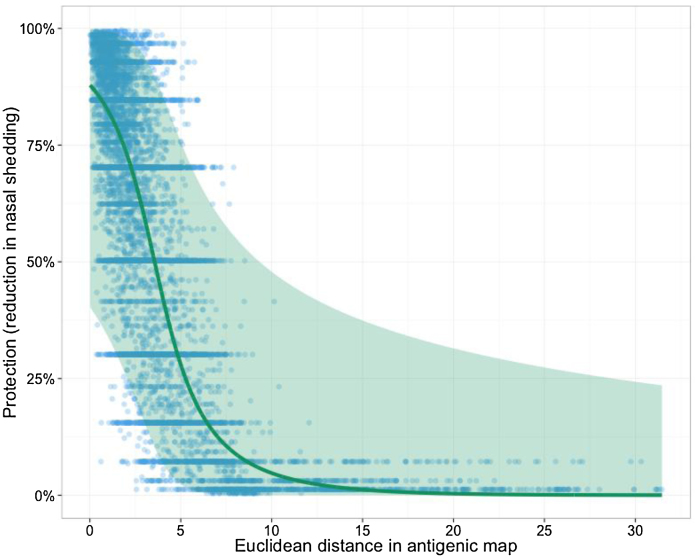
Relationship between distance in antigenic map and estimated protection. Bedford et al. [8] produced a map of H3N2 based on ∼10,000 measurements of the HI titre of ferret sera raised against a particular strain against different antigens. For each of these measurements we show (i) the Euclidean distance between the position of each sera and antigen in the map and (ii) the extent of protection (defined as reduction in nasal shedding) that we would predict based on the observed HI titre (blue points). We also show the results of a beta regression of map distance against protection (solid green line; shaded green area corresponds to 95% CI). (For interpretation of the references to color in this figure legend, the reader is referred to the web version of this article.)

**Fig. 5:**
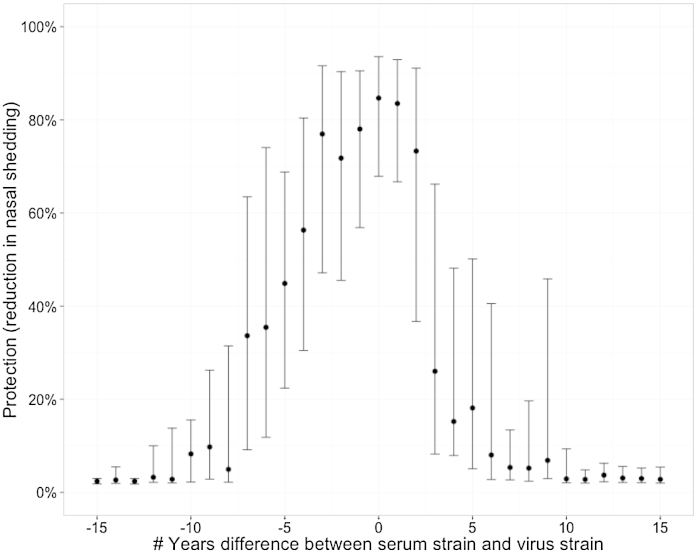
Protective effect through time. For each sera-antigen pair analysed in Bedford et al., this shows how the average protection (defined as reduction in nasal shedding) predicted by the HI titre of serum against antigen is affected by the of number of years difference between the date of isolation of the serum and the date of isolation of the antigen (positive numbers mean that the antigen was collected after the serum). Comparisons between sera and antigen that were isolated close together in time are much more common than between those isolated far apart in time and so this represents the median and 50% CI for predicted protection based on a bootstrap of 1000 samples for each measurement.

Generally, these results suggest that we should not treat fold-difference in homologous and heterologous titre (i.e. position within current antigenic maps) as the primary measure of the cross-protection of different influenza viruses. Instead, we should consider the absolute value of a heterologous titre. The importance of this concept is illustrated in Fig. 4: in maps where virus co-ordinates are derived from a function of fold-difference in titre, distance is a generally poor predictor of cross-protection. Of course, there is a correlation between the two as you would expect, but almost anything is possible for a distance of 5 units in the map. Further, as shown in Supplementary Figure S4, there is often extensive cross-protection between different antigenic clusters as defined in existing maps, and also between viruses isolated many years apart (Fig. 5). These results nevertheless support the concept that exposure to a currently circulating virus will provide very high levels of protection for the next 2–3 years, but the size of the confidence intervals means that we cannot rule out significant levels of protection over a much longer period.

## 4 Discussion

To try to improve our understanding of the antigenic evolution of the human influenza virus, we have estimated the effect of HI titre on both (i) the probability of a ferret exhibiting a serological response to infection and (ii) the reduction of virus produced in ferret nasal wash 3 days after challenge.

Both these measures should in some way correlate with the probability of onward transmission. Normally, a serological response is taken as evidence that a successful infection (i.e. viral replication) has taken place, and that (further) mobilisation of the adaptive immune system was required for its termination. Its absence is strongly suggestive that only limited viral replication took place, but the extent of this limitation (and its consequences for onward transmission) is poorly defined. The second measure considered here explicitly captures the extent of the restrictions imposed on the virus. Both measures suggest that there is a strong sigmoidal relationship between protection and HI titre and that it is the absolute value of a titre that determines the extent of protection. Thus, a doubled HI titre is not necessarily twice as biologically effective; this may also extend to other similar immunological assays.

Given that we routinely draw inferences about cross-protection and antigenic similarity between human influenza viruses based on the results of the HI assay in ferrets, it is imperative to understand the connection between ferret and human HI results. Both of the curves shown here are further to the right than the curve reported by Coudeville et al. for the prevention of a serological response in humans [9]; indeed they report a 50% protective titre against seroconversion of 17 (95% CI 10–29). It is probable that the inoculum used in the experiments on ferrets is much larger than that encountered by a human during an average seasonal exposure: thus much higher levels of circulating antibody may be required to prevent seroconversion (and infection more generally) in laboratory ferrets than in wild humans. A similar reason (high levels of virus shedding) was proposed to explain why, in a study protection against influenza infection in Hong Kong households [24], the 50% protective titre was found to be significantly higher than that observed by Coudeville et al. [9] (260 (95% CI 30–2009) for A/H3N2 and 255 (95% CI 62–917) for A/H1N1). However, it could also be that the RT-PCR analysis of nasal and throat swabs employed by [24] is more sensitive than seroconversion to influenza infection. Although an inverse relationship between the amount of virus produced and the likelihood of onward transmission is intuitive, there is no data to support the specific choice made here (that the percent reduction in viral titre on a log scale corresponds to an equivalent drop in infectiousness). A particular concern is that the amount of virus produced after 3 days may not be entirely representative of the virus's actual transmission potential. Experiments [25] with A(H1N1)pdm09 have shown that the amount of virus in ferret nasal wash is maximised 1–2 days after challenge. At this time, the animals are asymptomatic but nevertheless capable of onward transmission. The amount of virus in nasal wash decreases thereafter, and no onward transmission is possible by day 5 even though virus can be found in nasal wash and ferrets are symptomatic. Further, although the experiments of [25] used a different assay (the number of Plaque Forming Units per ml of nasal wash [PFU/ml]) to measure viral titre, its value on day 5 was still around 4log10 PFU/ml. Given that no transmission was observed at this time, this may in itself imply that there is a threshold amount of virus that must be produced for there to be any chance of onward transmission. If true, the simple relationship used here may be inappropriate to describe the relationship between viral titre and infectiousness.

A critical point with regard to this work's utility is that it provides a potential bridge from a single ferret exposure to a single human exposure. Of course, reality is much more complex: the natural human immune profile is dictated by repeat exposure to several influenza strains across the course of a lifetime. Furthermore, protection in both ferrets and humans is likely mediated by more than just whatever we measure in the HI assay [26,27]. This work does however emphasise that fairly low HI titres can be enough to indicate cross-protection. A consequence of this is that cross-protection can be observed across different antigenic clusters but how then do influenza viruses manage to cause regular epidemics, and why is vaccination with the previous year's dominantly circulating strain not more effective [28]? One possibility is that although vaccination of a *naïve* individual with last season's dominant strain may be enough to protect them against the next wave of viruses, the same may not be true in someone who is immunologically experienced. If pre-existing antibodies are enough to prevent replication of the vaccine strain, then there is no stimulus to produce a *de novo* response that could protect against the next antigenic cluster. This is a form of original antigenic sin [29] and is consistent with recently published results suggesting that vaccine effectiveness is higher in those who have not been recently vaccinated [30]. Similarly, perhaps the next wave of viruses are not selected for their ability to escape immunity arising from infection in the current epidemic, but rather for their ability to escape from immunity in individuals that was generated several epidemics previously [31].

One issue with the results presented here is that much of the data analysed in this study has come from ferrets on which various vaccines were being tested. It is likely that vaccinated individuals may exhibit HI titres that are inflated relative to their true level of protection [28], and, alongside a high challenge dose, this is another potential reason why (i) the curves we report here are further to the right than the equivalent in humans (as the ferrets are less protected than suggested by HI) and (ii) we observe significant variability among ferrets (as the precise effect could be vaccine-dependent). At the same time, since the type of vaccine may confound the use of seroconversion as a useful indicator of exposure [32], this study is strengthened by our simultaneous examination of viral titre in ferrets.

Another issue is that this data represents responses in ferrets solely to 1968 A/H3N2 and 1934 A/H1N1 viruses: it is conceivable that the response curves could vary among viruses or subtypes (e.g., see [33]). Nevertheless, there is not sufficient data available to test this here. In particular, although many papers present data on serological responses in ferrets, more recent authors tend to report this data as an average across similarly treated ferrets (usually 3). Yet, even though the mean and range of titres across 3 ferrets might be reported, the reported mean titre is often incompatible with a third measurement inside this range; thus these data cannot be analysed because they are not self-consistent. Coupled with information on the apparent variability of responses within individual ferrets, this emphasises a need to report data on individual ferrets alongside aggregated data in future.

Overall, this work shows that absolute HI titres are more important than relative ones for determining cross protection. However, there is an urgent need for more and better data – more measurements from ferrets with intermediate HI titres, and against a wider panel of viruses – to refine our understanding of this important relationship and better relate results in ferrets to outcomes in humans. Nor should we forget that other aspects of the immune response will contribute to protection in both.

## Figures and Tables

**Table 1:** Parameter estimates for model of effect of HI titre on probability of a serological response.

Quantiles	Parameter estimate		
	*χ*	*Φ*	*P*
2.5%	7.41	0.71	0.95
50%	8.23	1.23	0.99
97.5%	8.99	2.15	1.00

**Table 2:** Parameter estimates for model of effect of HI titre on reduction of virus production in undiluted nasal wash.

Quantiles	Parameter estimate			
	*α*	*β*	*γ*	*σ*
2.5%	6.62	0.54	4.76	0.89
50%	7.31	0.85	4.98	1.01
97.5%	7.83	1.00	5.07	1.06
