# Supplementary material for: Relationship between haemagglutination inhibition titre and immunity to influenza in ferrets
Source: Vaccine. 2015 Oct 5;33(41):5380–5. doi: 10.1016/j.vaccine.2015.08.065 (PMC4582772; doi:10.1016/j.vaccine.2015.08.065)
Supplement: Supplementary file 1 [file mmc1.docx]

**Supplemental File**

**Figure S1**

**
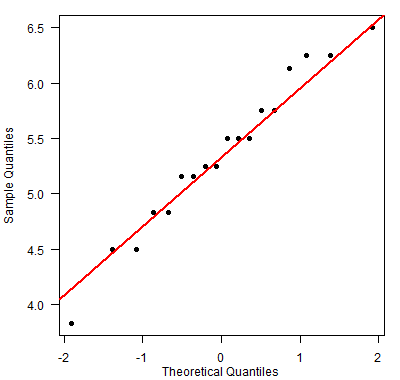
**

**Q-Q plot of virus production in naive ferrets against a normal distribution.** This shows that the quantiles of the normal distribution are similar to those of the empirical distribution of log_10_ virus titre in naive ferrets, hence justifying the choice of modelling viral titre in ferrets as lognormally distributed. The black dots represent the individual data points, whilst the red line gives the the line on which these points should lie if they are normally distributed.

**Figure S2**


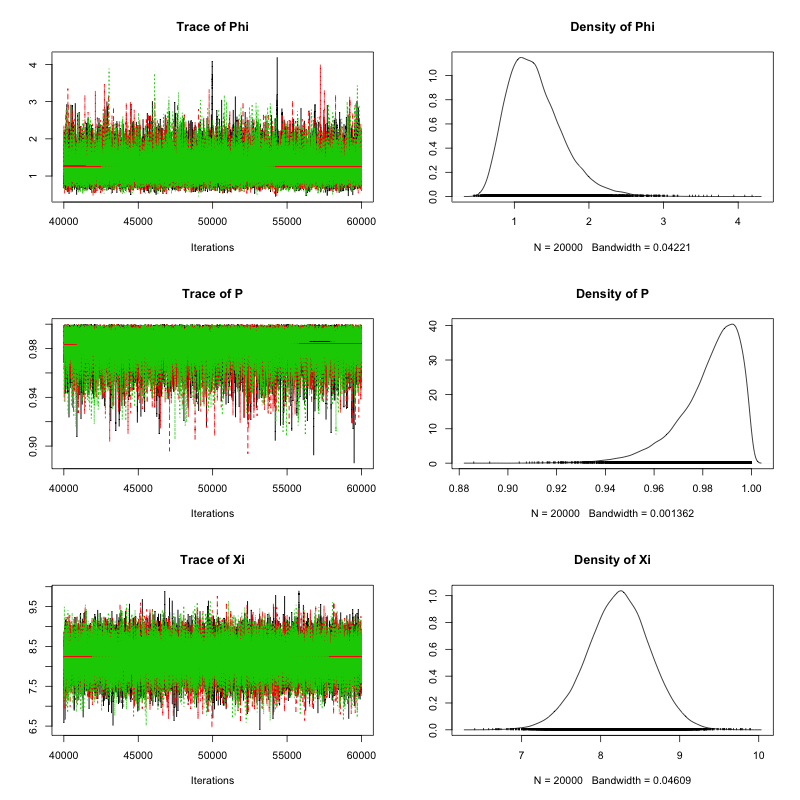


**Trace and density plots of parameters used in model for effect of pre-existing HI titre on probability of a serological response**

**Figure S3**


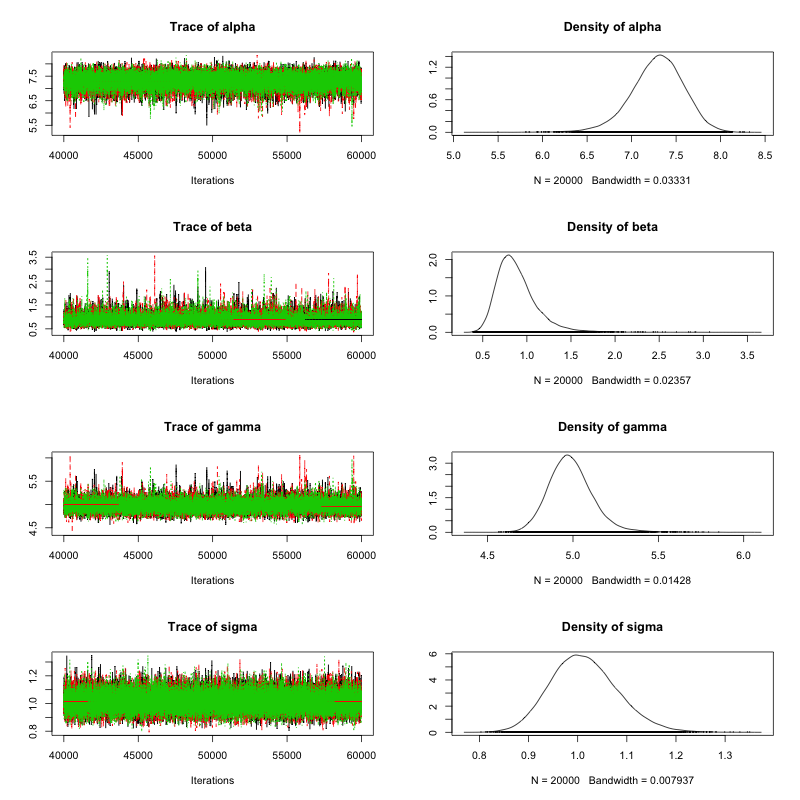


**Trace and density plots of parameters used in model for effect of pre-existing HI titre on reduction of virus production in undiluted nasal wash**

**Figure S4**

**
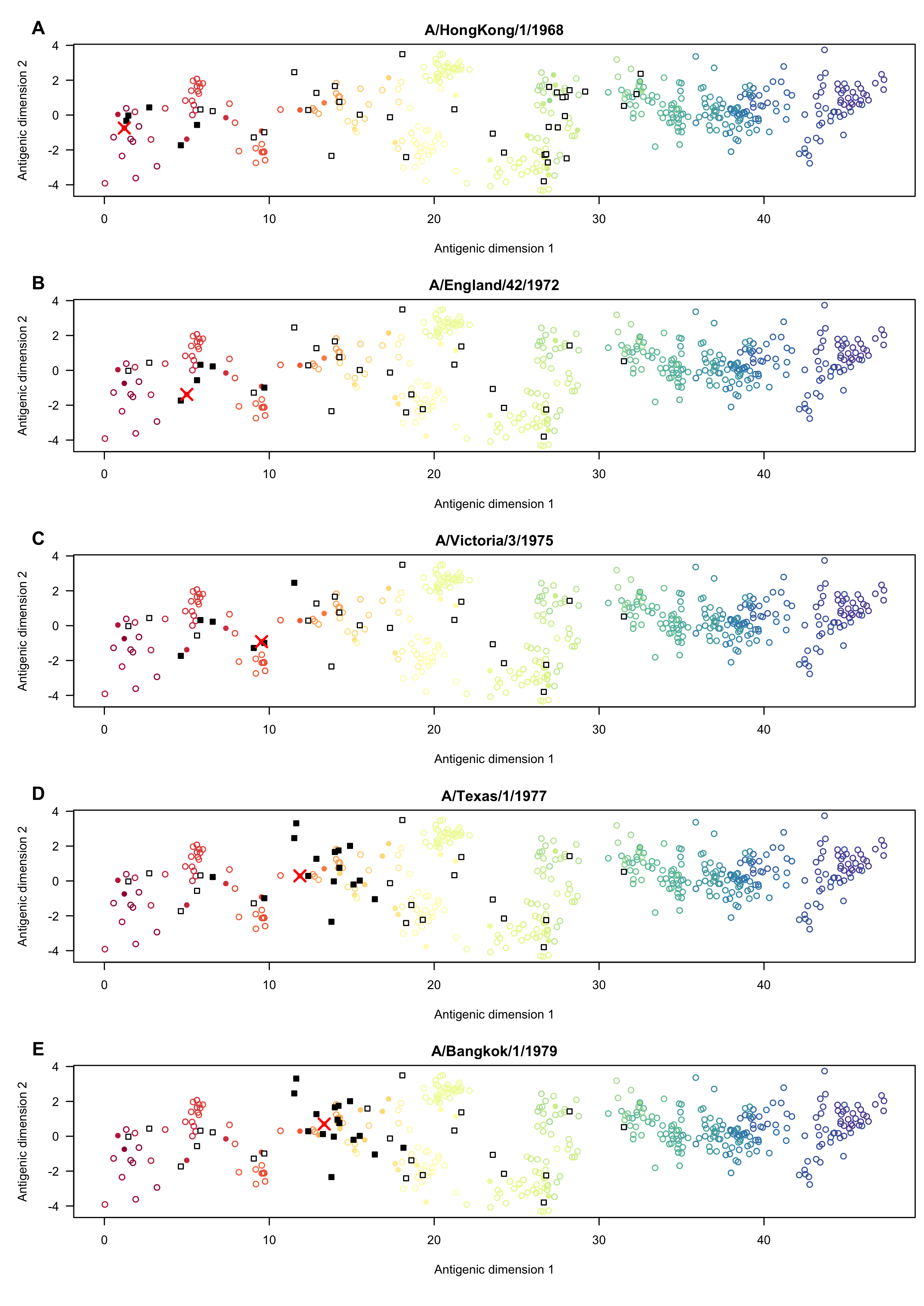
**

**
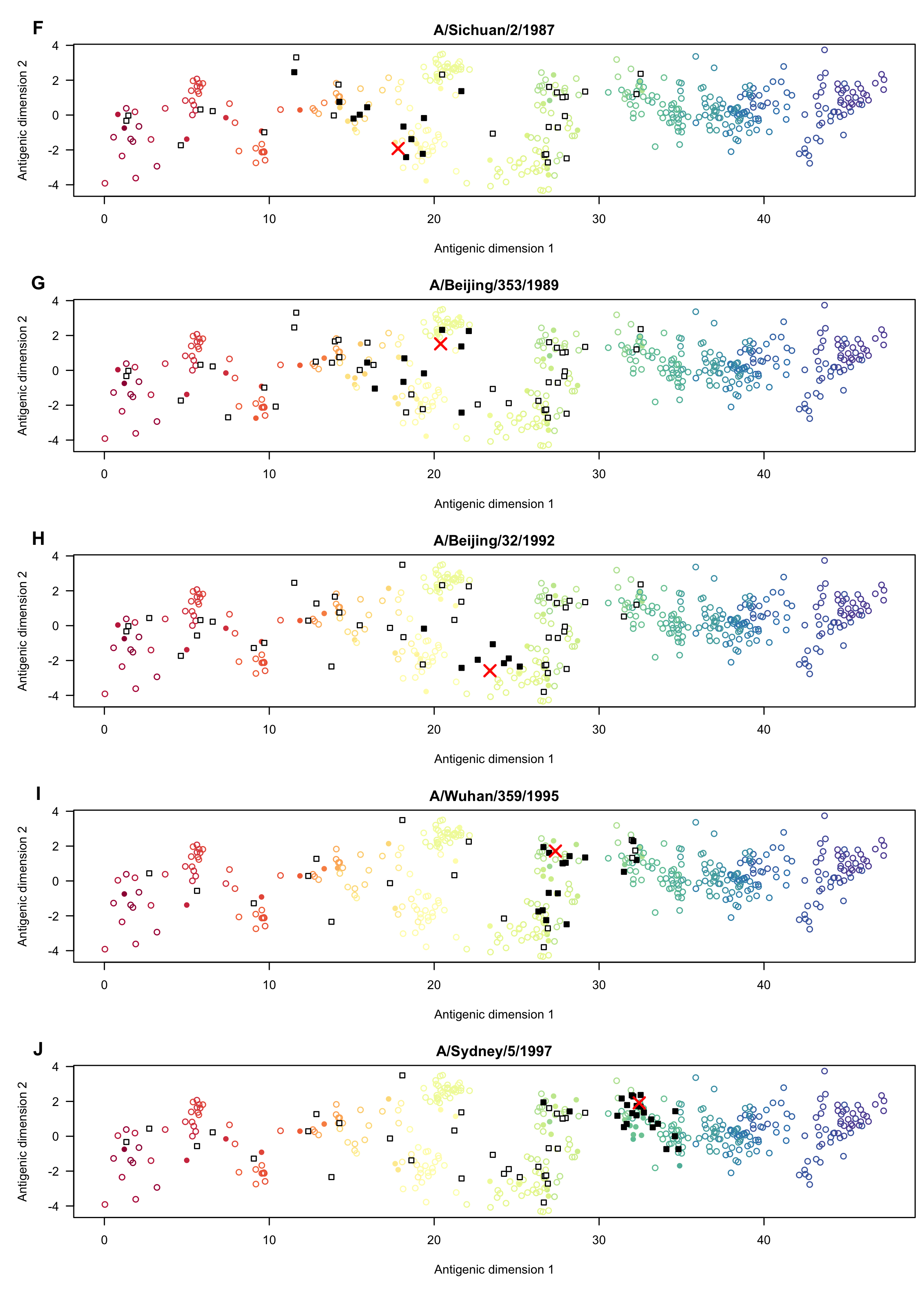
**

**
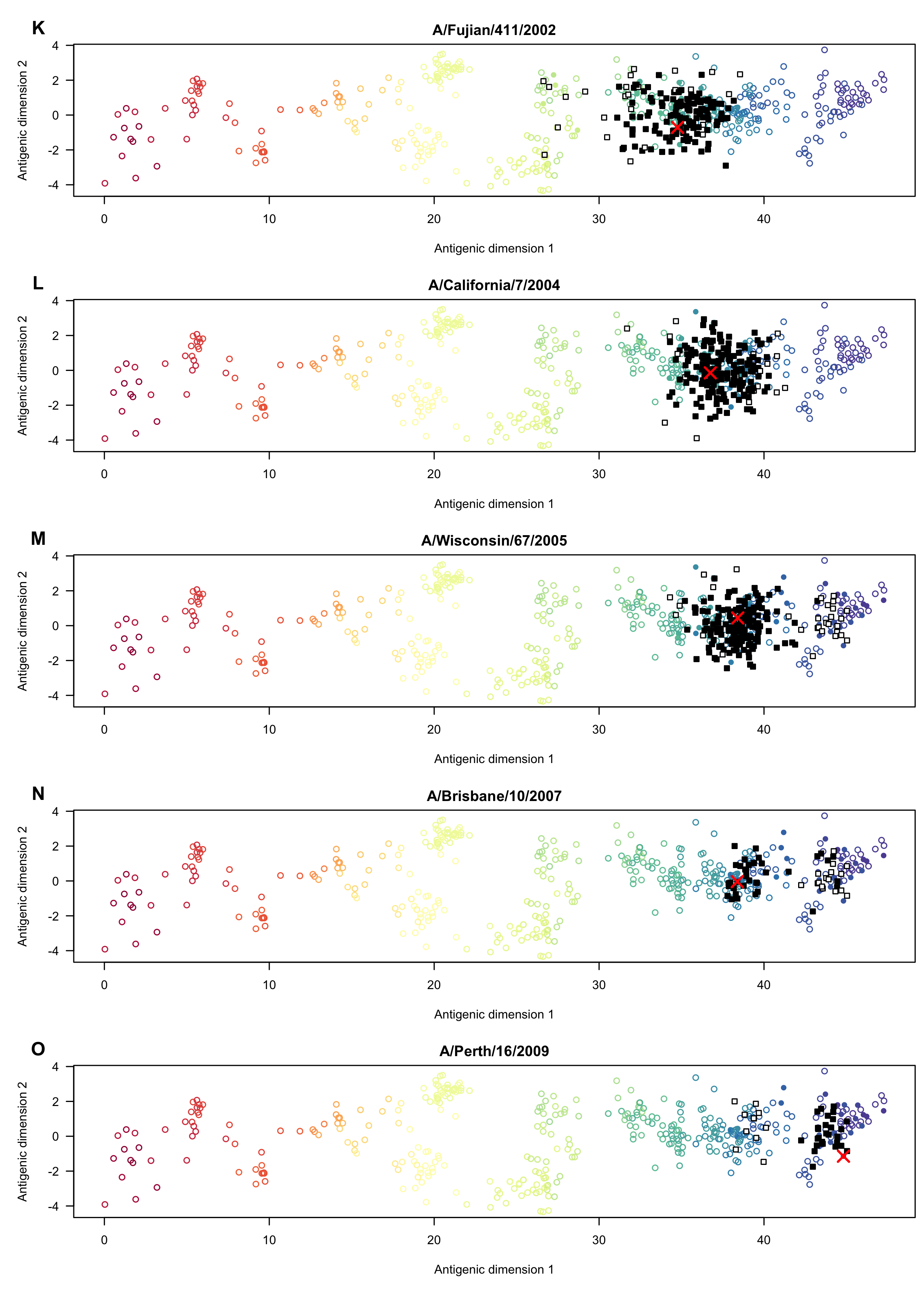
**

**Superimposing predicted protection on antigenic maps.** Each of these figures consists of the antigenic map of H3N2 virus isolates presented by Bedford et al. [8] but with different aspects highlighted. Open coloured circles indicate the position of each virus isolate, whose colour corresponds to the year of isolation (red = 1968 through to blue = 2009). A red cross highlights the location of a single H3N2 virus within each map, and there is one map for each of the canonical cluster-defining variants of H3N2 (labelled above each map). Squares then represent the locations of ferret sera that have been used in HI assays against that virus. The circles of the viral isolates that correspond to these sera are then filled in, whilst the squares themselves are coloured white if the HI titre would predict <50% protection (measured in terms of a reduction in the titre of virus in ferret nasal wash), and black otherwise.
