# Supplementary material for: Relationship between haemagglutination inhibition titre and immunity to influenza in ferrets
Source: Vaccine. 2015 Oct 5;33(41):5380–5. doi: 10.1016/j.vaccine.2015.08.065 (PMC4582772; doi:10.1016/j.vaccine.2015.08.065)
Supplement: Supplementary file 2 [file mmc2.pdf]

Ferret Ref	Treatment 1						Post Treatment Virus Isolation			Treatment 2						Pre challenge		Challenge				Post challenge			Post Challenge Virus Isolation			Paper			
Study	Pre Treatment HI (if measured)	Virus	Subtype	Type	Dose	Route	Days Post Treatment	Type	Titre	Days Post .Treatment 1	Virus	Subtype	Type	Dose	Route	HI titre	Days Post Last Treatment	Virus	Subtype	Dose	Days Post Last Treatment	HI titre	Days Post Challenge	Serologic Response?	Days Post Challenge	Type	Titre	Ref #	Lead Author	Pub Year	PMID
278		HK/1/68	H3N2	Infection	10^6-7 EID 50	IN	3	Nasal Wash	5.16							2560	32	HK/1/68	H3N2	10^7 EID 50	35	1976	21	N	3	Nasal Wash	0.00	19	McLaren	1974	4422499
279		HK/1/68	H3N2	Infection	10^6-7 EID 50	IN	3	Nasal Wash	5.16							>5120	32	HK/1/68	H3N2	10^7 EID 50	35	2560	21	N	3	Nasal Wash	0.00	19	McLaren	1974	4422499
281		HK/1/68	H3N2	Infection	10^6-7 EID 50	IN	3	Nasal Wash	4.5							2560	32	HK/1/68	H3N2	10^7 EID 50	35	5120	21	N	3	Nasal Wash	0.00	19	McLaren	1974	4422499
285		PR/8/34	H3N2	Infection	10^6-7 EID 50	IN	3	Nasal Wash	4.16							<5	32	HK/1/68	H3N2	10^7 EID 50	35	30	21	Y	3	Nasal Wash	2.50	19	McLaren	1974	4422499
286		PR/8/34	H3N2	Infection	10^6-7 EID 50	IN	3	Nasal Wash	5.16							5	32	HK/1/68	H3N2	10^7 EID 50	35	>5120	21	Y	3	Nasal Wash	4.13	19	McLaren	1974	4422499
287		PR/8/34	H3N2	Infection	10^6-7 EID 50	IN	3	Nasal Wash	5.16							<5	32	HK/1/68	H3N2	10^7 EID 50	35	>5120	21	Y	3	Nasal Wash	2.13	19	McLaren	1974	4422499
288		PR/8/34	H3N2	Infection	10^6-7 EID 50	IN	3	Nasal Wash	5.5							<5	32	HK/1/68	H3N2	10^7 EID 50	35	5120	21	Y	3	Nasal Wash	4.13	19	McLaren	1974	4422499
289		PR/8/34	H3N2	Infection	10^6-7 EID 50	IN	3	Nasal Wash	5.16							<5	32	HK/1/68	H3N2	10^7 EID 50	35	1280	21	Y	3	Nasal Wash	4.13	19	McLaren	1974	4422499
290		64b	H1N2	Infection	10^6-7 EID 50	IN	3	Nasal Wash	5.16							<5	32	HK/1/68	H3N2	10^7 EID 50	35	>5120	21	Y	3	Nasal Wash	3.83	19	McLaren	1974	4422499
291		64b	H1N2	Infection	10^6-7 EID 50	IN	3	Nasal Wash	4.5							<5	32	HK/1/68	H3N2	10^7 EID 50	35	1280	21	Y	3	Nasal Wash	3.50	19	McLaren	1974	4422499
292		64b	H1N2	Infection	10^6-7 EID 50	IN	3	Nasal Wash	4.5							<5	32	HK/1/68	H3N2	10^7 EID 50	35	40	21	Y	3	Nasal Wash	4.50	19	McLaren	1974	4422499
293		64b	H1N2	Infection	10^6-7 EID 50	IN	3	Nasal Wash	4.83							<5	32	HK/1/68	H3N2	10^7 EID 50	35	640	21	Y	3	Nasal Wash	3.83	19	McLaren	1974	4422499
301		64b	H1N2	Infection	10^6-7 EID 50	IN	3	Nasal Wash	4.83							<5	32	HK/1/68	H3N2	10^7 EID 50	35	1280	21	Y	3	Nasal Wash	2.83	19	McLaren	1974	4422499
302		31a	H3N1	Infection	10^6-7 EID 50	IN	3	Nasal Wash	4.5							5120	32	HK/1/68	H3N2	10^7 EID 50	35	1280	21	N	3	Nasal Wash	0.00	19	McLaren	1974	4422499
304		31a	H3N1	Infection	10^6-7 EID 50	IN	3	Nasal Wash	5.16							2560	32	HK/1/68	H3N2	10^7 EID 50	35	1920	21	N	3	Nasal Wash	0.00	19	McLaren	1974	4422499
305		31a	H3N1	Infection	10^6-7 EID 50	IN	3	Nasal Wash	5.16							960	32	HK/1/68	H3N2	10^7 EID 50	35	NR	21	NA	3	Nasal Wash	0.00	19	McLaren	1974	4422499
308		31a	H3N1	Infection	10^6-7 EID 50	IN	3	Nasal Wash	3.83							240	32	HK/1/68	H3N2	10^7 EID 50	35	960	21	Y	3	Nasal Wash	1.60	19	McLaren	1974	4422499
283		NONE														<5		HK/1/68	H3N2	10^7 EID 50	35	1280	21	Y	3	Nasal Wash	6.13	19	McLaren	1974	4422499
284		NONE														<5		HK/1/68	H3N2	10^7 EID 50	35	2560	21	Y	3	Nasal Wash	3.83	19	McLaren	1974	4422499
295		NONE														<5		HK/1/68	H3N2	10^7 EID 50	35	2560	21	Y	3	Nasal Wash	4.50	19	McLaren	1974	4422499
296		NONE														<5		HK/1/68	H3N2	10^7 EID 50	35	<5	21	N	3	Nasal Wash	4.83	19	McLaren	1974	4422499
309		NONE														<5		HK/1/68	H3N2	10^7 EID 50	35	1920	21	Y	3	Nasal Wash	6.50	19	McLaren	1974	4422499
311		NONE														<5		HK/1/68	H3N2	10^7 EID 50	35	960	21	Y	3	Nasal Wash	5.50	19	McLaren	1974	4422499
261		HK/1/68	H3N2	Infection	10^6-7 EID 50	IN										<5	25	PR/8/34	H1N1	10^6-7 EID 50	28	2560	14	Y	3	Nasal Wash	4.16	19	McLaren	1974	4422499
260		HK/1/68	H3N2	Infection	10^6-7 EID 50	IN										<5	25	PR/8/34	H1N1	10^6-7 EID 50	28	480	14	Y	3	Nasal Wash	6.16	19	McLaren	1974	4422499
253		HK/1/68	H3N2	Infection	10^6-7 EID 50	IN										<5	25	PR/8/34	H1N1	10^6-7 EID 50	28	>5120	14	Y	3	Nasal Wash	6.51	19	McLaren	1974	4422499
258		HK/1/68	H3N2	Infection	10^6-7 EID 50	IN										<5	25	PR/8/34	H1N1	10^6-7 EID 50	28	2560	14	Y	3	Nasal Wash	5.16	19	McLaren	1974	4422499
249		HK/1/68	H3N2	Infection	10^6-7 EID 50	IN										<5	25	64b	H1N2	10^6-7 EID 50	28	240	14	Y	3	Nasal Wash	0.50	19	McLaren	1974	4422499
252		HK/1/68	H3N2	Infection	10^6-7 EID 50	IN										<5	25	64b	H1N2	10^6-7 EID 50	28	<5	14	N	3	Nasal Wash	0.50	19	McLaren	1974	4422499
256		HK/1/68	H3N2	Infection	10^6-7 EID 50	IN										<5	25	64b	H1N2	10^6-7 EID 50	28	640	14	Y	3	Nasal Wash	2.00	19	McLaren	1974	4422499
264		HK/1/68	H3N2	Infection	10^6-7 EID 50	IN										<5	25	64b	H1N2	10^6-7 EID 50	28	960	14	Y	3	Nasal Wash	2.83	19	McLaren	1974	4422499
247		HK/1/68	H3N2	Infection	10^6-7 EID 50	IN										1200	25	HK/1/68	H3N2	10^6-7 EID 50	28	800	14	N	3	Nasal Wash	0.00	19	McLaren	1974	4422499
250		HK/1/68	H3N2	Infection	10^6-7 EID 50	IN										3200	25	HK/1/68	H3N2	10^6-7 EID 50	28	800	14	N	3	Nasal Wash	0.00	19	McLaren	1974	4422499
254		HK/1/68	H3N2	Infection	10^6-7 EID 50	IN										2400	25	HK/1/68	H3N2	10^6-7 EID 50	28	1200	14	N	3	Nasal Wash	0.00	19	McLaren	1974	4422499
262		HK/1/68	H3N2	Infection	10^6-7 EID 50	IN										400	25	HK/1/68	H3N2	10^6-7 EID 50	28	600	14	N	3	Nasal Wash	0.00	19	McLaren	1974	4422499
25		HK/1/68	H3N2	Infection	10^6-7 EID 50	IN										1280	25	31a	H3N1	10^6-7 EID 50	28	1280	14	N	3	Nasal Wash	0.00	19	McLaren	1974	4422499
32		HK/1/68	H3N2	Infection	10^6-7 EID 50	IN										2560	25	31a	H3N1	10^6-7 EID 50	28	2560	14	N	3	Nasal Wash	0.00	19	McLaren	1974	4422499
135		Aichi/2/68	H3N2	Vaccine x2 dose	150 CCA	IN										<5	35	HK/1/68	H3N2	3*10^6EID50		3200	21	Y	3	Nasal Wash	5.25	18	McLaren	1974	4830530
138		Aichi/2/68	H3N2	Vaccine x2 dose	150 CCA	IN										<5	35	HK/1/68	H3N2	3*10^6EID50		2400	21	Y	3	Nasal Wash	4.75	18	McLaren	1974	4830530
140		Aichi/2/68	H3N2	Vaccine x2 dose	150 CCA	IN										<5	35	HK/1/68	H3N2	3*10^6EID50		1200	21	Y	3	Nasal Wash	6.25	18	McLaren	1974	4830530
141		Aichi/2/68	H3N2	Vaccine x2 dose	150 CCA	IN										<5	35	HK/1/68	H3N2	3*10^6EID50		1200	21	Y	3	Nasal Wash	5.50	18	McLaren	1974	4830530
129		Aichi/2/68	H3N2	Vaccine x4 dose	150 CCA	IN										<5	35	HK/1/68	H3N2	3*10^6EID50		2400	21	Y	3	Nasal Wash	4.50	18	McLaren	1974	4830530
130		Aichi/2/68	H3N2	Vaccine x4 dose	150 CCA	IN										<5	35	HK/1/68	H3N2	3*10^6EID50		600	21	Y	3	Nasal Wash	5.25	18	McLaren	1974	4830530
131		Aichi/2/68	H3N2	Vaccine x4 dose	150 CCA	IN										<5	35	HK/1/68	H3N2	3*10^6EID50		1200	21	Y	3	Nasal Wash	5.75	18	McLaren	1974	4830530
132		Aichi/2/68	H3N2	Vaccine x4 dose	150 CCA	IN										<5	35	HK/1/68	H3N2	3*10^6EID50		1200	21	Y	3	Nasal Wash	5.50	18	McLaren	1974	4830530
142		NONE														<5	35	HK/1/68	H3N2	3*10^6EID50		800	21	Y	3	Nasal Wash	5.75	18	McLaren	1974	4830530
143		NONE														<5	35	HK/1/68	H3N2	3*10^6EID50		1600	21	Y	3	Nasal Wash	6.25	18	McLaren	1974	4830530
144		NONE														<5	35	HK/1/68	H3N2	3*10^6EID50		2400	21	Y	3	Nasal Wash	5.25	18	McLaren	1974	4830530
145		NONE														<5	35	HK/1/68	H3N2	3*10^6EID50		800	21	Y	3	Nasal Wash	5.50	18	McLaren	1974	4830530
418		PR/8/34	H1N1	Infection						35	HK/1/68	H3N2	Vaccine	400 IU on day 0 + 200 IU on day 1	IN	60	12	HK/1/68	H3N2	3*10^6EID50	12	640	21	Y	3	Nasal Wash	4.50	18	McLaren	1974	4830530
419		PR/8/34	H1N1	Infection						35	HK/1/68	H3N2	Vaccine	400 IU on day 0 + 200 IU on day 1	IN	30	12	HK/1/68	H3N2	3*10^6EID50	12	240	21	Y	3	Nasal Wash	5.16	18	McLaren	1974	4830530
420		PR/8/34	H1N1	Infection						35	HK/1/68	H3N2	Vaccine	400 IU on day 0 + 200 IU on day 1	IN	120	12	HK/1/68	H3N2	3*10^6EID50	12	120	21	N	3	Nasal Wash	3.50	18	McLaren	1974	4830530
421		PR/8/34	H1N1	Infection						35	HK/1/68	H3N2	Vaccine	400 IU on day 0 + 200 IU on day 1	IN	60	12	HK/1/68	H3N2	3*10^6EID50	12	3840	21	Y	3	Nasal Wash	5.50	18	McLaren	1974	4830530
437		PR/8/34	H1N1	Infection						35	HK/1/68	H3N2	Vaccine	200 IU on days 0,1,7,8	IN	15	12	HK/1/68	H3N2	3*10^6EID50	12	480	21	Y	3	Nasal Wash	1.83	18	McLaren	1974	4830530
438		PR/8/34	H1N1	Infection						35	HK/1/68	H3N2	Vaccine	200 IU on days 0,1,7,8	IN	240	12	HK/1/68	H3N2	3*10^6EID50	12	120	21	N	3	Nasal Wash	2.50	18	McLaren	1974	4830530
439		PR/8/34	H1N1	Infection						35	HK/1/68	H3N2	Vaccine	200 IU on days 0,1,7,8	IN	60	12	HK/1/68	H3N2	3*10^6EID50	12	240	21	Y	3	Nasal Wash	2.83	18	McLaren	1974	4830530
440		PR/8/34	H1N1	Infection						35	HK/1/68	H3N2	Vaccine	200 IU on days 0,1,7,8	IN	120	12	HK/1/68	H3N2	3*10^6EID50	12	240	21	N	3	Nasal Wash	0.00	18	McLaren	1974	4830530
424		PR/8/34	H1N1	Infection						35	HK/1/68	H3N2	Vaccine	400 IU on day 0	IM	2560	12	HK/1/68	H3N2	3*10^6EID50	12	480	21	N	3	Nasal Wash	0.00	18	McLaren	1974	4830530
425		PR/8/34	H1N1	Infection						35	HK/1/68	H3N2	Vaccine	400 IU on day 0	IM	240	12	HK/1/68	H3N2	3*10^6EID50	12	480	21	N	3	Nasal Wash	0.00	18	McLaren	1974	4830530
426		PR/8/34	H1N1	Infection						35	HK/1/68	H3N2	Vaccine	400 IU on day 0	IM	480	12	HK/1/68	H3N2	3*10^6EID50	12	60	21	N	3	Nasal Wash	0.00	18	McLaren	1974	4830530
427		PR/8/34	H1N1	Infection						35	HK/1/68	H3N2	Vaccine	720 IU on day 0 + 720 IU on day 1	IN	<10	14	HK/1/68	H3N2	3*10^6EID50	14	30	21	NA	3	Nasal Wash	4.83	18	McLaren	1974	4830530
428		PR/8/34	H1N1	Infection						35	HK/1/68	H3N2	Vaccine	720 IU on day 0 + 720 IU on day 1	IN	<10	14	HK/1/68	H3N2	3*10^6EID50	14	80	21	Y	3	Nasal Wash	4.50	18	McLaren	1974	4830530
429		PR/8/34	H1N1	Infection						35	HK/1/68	H3N2	Vaccine	720 IU on day 0 + 720 IU on day 1	IN	<10	14	HK/1/68	H3N2	3*10^6EID50	14	120	21	Y	3	Nasal Wash	3.83	18	McLaren	1974	4830530
430		PR/8/34	H1N1	Infection						35	HK/1/68	H3N2	Vaccine	720 IU on day 0 + 720 IU on day 1	IN	<10	14	HK/1/68	H3N2	3*10^6EID50	14	80	21	Y	3	Nasal Wash	4.83	18	McLaren	1974	4830530
431		PR/8/34	H1N1	Infection						35	HK/1/68	H3N2	Vaccine	720 IU on days 0,1,7,8	IN	<10	14	HK/1/68	H3N2	3*10^6EID50	14	160	21	Y	3	Nasal Wash	4.50	18	McLaren	1974	4830530
432		PR/8/34	H1N1	Infection						35	HK/1/68	H3N2	Vaccine	720 IU on days 0,1,7,8	IN	<10	14	HK/1/68	H3N2	3*10^6EID50	14	60	21	Y	3	Nasal Wash	4.50	18	McLaren	1974	4830530
433		PR/8/34	H1N1	Infection						35	HK/1/68	H3N2	Vaccine	720 IU on days 0,1,7,8	IN	<10	14	HK/1/68	H3N2	3*10^6EID50	14	640	21	Y	3	Nasal Wash	2.50	18	McLaren	1974	4830530
436		PR/8/34	H1N1	Infection						35	HK/1/68	H3N2	Vaccine	720 IU on days 0,1,7,8	IN	<10	14	HK/1/68	H3N2	3*10^6EID50	14	120	21	Y	3	Nasal Wash	3.83	18	McLaren	1974	4830530
25		HK/1/68	H3N2	Vaccine	720 IU on days 0,1,7,8	IN										<10	14	HK/1/68	H3N2	3*10^6EID50	14	1280	21	Y	3	Nasal Wash	5.50	18	McLaren	1974	4830530
26		HK/1/68	H3N2	Vaccine	720 IU on days 0,1,7,8	IN										<10	14	HK/1/68	H3N2	3*10^6EID50	14	2560	21	Y	3	Nasal Wash	4.16	18	McLaren	1974	4830530
32		HK/1/68	H3N2	Vaccine	720 IU on days 0,1,7,8	IN										<10	14	HK/1/68	H3N2	3*10^6EID50	14	1280	21	Y	3	Nasal Wash	6.50	18	McLaren	1974	4830530
104	<5	HK/3/68	H3N2	Infection	10^6.5 EID50		3	Nasal Wash	5.25							1280	33-34	HK/3/68	H3N2	10^6.5 EID50	35	480	28	N	3	Nasal Wash	0.00	17	Potter	1972	5032092
105	<5	HK/3/68	H3N2	Infection	10^6.5 EID50		3	Nasal Wash	5.25							1280	33-34	HK/3/68	H3N2	10^6.5 EID50	35	480	28	N	3	Nasal Wash	0.00	17	Potter	1972	5032092

88	<5	HK/3/68	H3N2	Infection	10^6.5 EID50											960	33-34	HK/3/68	H3N2	10^6.5 EID50	35	240	28	N	3	Nasal Wash	0.00	17	Potter	1972	5032092
106	<5	HK/3/68	H3N2	Infection	10^6.5 EID50		3	Nasal Wash	4.25							2560	33-34	HK/3/68	H3N2	10^6.5 EID50	35	480	28	N	3	Nasal Wash	0.00	17	Potter	1972	5032092
89	<5	HK/3/68	H3N2	Infection	10^6.5 EID50											1280	33-34	HK/3/68	H3N2	10^6.5 EID50	35	480	28	N	3	Nasal Wash	0.00	17	Potter	1972	5032092
99	<5	HK/1/68	H3N2	Infection	10^6.5 EID50		3	Nasal Wash	3.75							320	33-34	HK/3/68	H3N2	10^6.5 EID50	35	480	28	N	3	Nasal Wash	0.00	17	Potter	1972	5032092
100	<5	HK/1/68	H3N2	Infection	10^6.5 EID50		3	Nasal Wash	4.25							320	33-34	HK/3/68	H3N2	10^6.5 EID50	35	480	28	N	3	Nasal Wash	0.00	17	Potter	1972	5032092
101	<5	HK/1/68	H3N2	Infection	10^6.5 EID50		3	Nasal Wash	4.25							640	33-34	HK/3/68	H3N2	10^6.5 EID50	35	480	28	N	3	Nasal Wash	0.00	17	Potter	1972	5032092
102	<5	HK/1/68	H3N2	Infection	10^6.5 EID50		3	Nasal Wash	3.25							240	33-34	HK/3/68	H3N2	10^6.5 EID50	35	480	28	N	3	Nasal Wash	0.00	17	Potter	1972	5032092
91		B/Eng/13/65	B	Infection	10^6.5 EID50											<5	33-34	HK/3/68	H3N2	10^6.5 EID50	35	640	28	Y	3	Nasal Wash	4.75	17	Potter	1972	5032092
92		B/Eng/13/65	B	Infection	10^6.5 EID50											<5	33-34	HK/3/68	H3N2	10^6.5 EID50	35	480	28	Y	3	Nasal Wash	5.50	17	Potter	1972	5032092
93		B/Eng/13/65	B	Infection	10^6.5 EID50											<5	33-34	HK/3/68	H3N2	10^6.5 EID50	35	480	28	Y	3	Nasal Wash	5.25	17	Potter	1972	5032092
94		B/Eng/13/65	B	Infection	10^6.5 EID50											<5	33-34	HK/3/68	H3N2	10^6.5 EID50	35	480	28	Y	3	Nasal Wash	4.75	17	Potter	1972	5032092
90		"HK vaccine"	H3N2	Vaccine	8000 HAU											<5	28	HK/3/68	H3N2	10^6.5 EID50	35	480	28	Y	3	Nasal Wash	4.75	17	Potter	1972	5032092
95		"HK vaccine"	H3N2	Vaccine	8000 HAU											<5	28	HK/3/68	H3N2	10^6.5 EID50	35	960	28	Y	3	Nasal Wash	4.50	17	Potter	1972	5032092
96		"HK vaccine"	H3N2	Vaccine	8000 HAU											<5	28	HK/3/68	H3N2	10^6.5 EID50	35	960	28	Y	3	Nasal Wash	5.50	17	Potter	1972	5032092
97		"HK vaccine"	H3N2	Vaccine	8000 HAU											80	28	HK/3/68	H3N2	10^6.5 EID50	35	1920	28	Y	3	Nasal Wash	0.00	17	Potter	1972	5032092
110		"HK vaccine"	H3N2	Vaccine	8000 HAU											<5	28	HK/3/68	H3N2	10^6.5 EID50	35	240	28	Y	3	Nasal Wash	5.50	17	Potter	1972	5032092
111		"HK vaccine"	H3N2	Vaccine	8000 HAU											<5	28	HK/3/68	H3N2	10^6.5 EID50	35	240	28	Y	3	Nasal Wash	5.25	17	Potter	1972	5032092
115		"HK vaccine"	H3N2	Vaccine	8000 HAU											<5	28	HK/3/68	H3N2	10^6.5 EID50	35	640	28	Y	3	Nasal Wash	5.75	17	Potter	1972	5032092
174		NONE														<5		HK/3/68	H3N2	10^6.5 EID50	35	800	32-34	Y	3	Nasal Wash	5.25	16	Potter	1973	4511952
175		NONE														<5		HK/3/68	H3N2	10^6.5 EID50	35	1600	32-34	Y	3	Nasal Wash	5.50	16	Potter	1973	4511952
176		NONE														<5		HK/3/68	H3N2	10^6.5 EID50	35	2400	32-34	Y	3	Nasal Wash	6.25	16	Potter	1973	4511952
177		NONE														<5		HK/3/68	H3N2	10^6.5 EID50	35	800	32-34	Y	3	Nasal Wash	5.75	16	Potter	1973	4511952
162		Aichi/2/68	H3N2	Killed Vaccine	400 CCA	IM										<5	35	HK/3/68	H3N2	10^6.5 EID50	35	800	32-34	Y	3	Nasal Wash	5.25	16	Potter	1973	4511952
170		Aichi/2/68	H3N2	Killed Vaccine	400 CCA	IM										<5	35	HK/3/68	H3N2	10^6.5 EID50	35	600	32-34	Y	3	Nasal Wash	5.75	16	Potter	1973	4511952
171		Aichi/2/68	H3N2	Killed Vaccine	400 CCA	IM										<5	35	HK/3/68	H3N2	10^6.5 EID50	35	300	32-34	Y	3	Nasal Wash	5.50	16	Potter	1973	4511952
172		Aichi/2/68	H3N2	Killed Vaccine	400 CCA	IM										15	35	HK/3/68	H3N2	10^6.5 EID50	35	1200	32-34	Y	3	Nasal Wash	5.50	16	Potter	1973	4511952
163		Aichi/2/68	H3N2	Killed Vaccine+Adj	400 CCA	IM										800	35	HK/3/68	H3N2	10^6.5 EID50	35	1600	32-34	N	3	Nasal Wash	2.75	16	Potter	1973	4511952
164		Aichi/2/68	H3N2	Killed Vaccine+Adj	400 CCA	IM										1200	35	HK/3/68	H3N2	10^6.5 EID50	35	1600	32-34	N	3	Nasal Wash	0.00	16	Potter	1973	4511952
165		Aichi/2/68	H3N2	Killed Vaccine+Adj	400 CCA	IM										400	35	HK/3/68	H3N2	10^6.5 EID50	35	600	32-34	N	3	Nasal Wash	3.25	16	Potter	1973	4511952
166		Aichi/2/68	H3N2	Killed Vaccine+Adj	400 CCA	IM										1200	35	HK/3/68	H3N2	10^6.5 EID50	35	1200	32-34	N	3	Nasal Wash	<1.5	16	Potter	1973	4511952
326		B/AA/66	B	Infection		IN				35	HK/X31/68	H3N2	Vaccine	200 IU	IM	<10	35	HK/1/68	H3N2	10^7 EID50	35	1280	21	Y	3	Nasal Wash	5.16	15	McLaren	1974	4522247
327		B/AA/66	B	Infection		IN				35	HK/X31/68	H3N2	Vaccine	200 IU	IM	<10	35	HK/1/68	H3N2	10^7 EID50	35	960	21	Y	3	Nasal Wash	4.83	15	McLaren	1974	4522247
328		B/AA/66	B	Infection		IN				35	HK/X31/68	H3N2	Vaccine	200 IU	IM	<10	35	HK/1/68	H3N2	10^7 EID50	35	640	21	Y	3	Nasal Wash	4.16	15	McLaren	1974	4522247
330		B/AA/66	B	Infection		IN				35	HK/X31/68	H3N2	Vaccine	200 IU	IM	<10	35	HK/1/68	H3N2	10^7 EID50	35	960	21	Y	3	Nasal Wash	5.16	15	McLaren	1974	4522247
324		PR/8/34	H1N1	Infection		IN				35	HK/X31/68	H3N2	Vaccine	200 IU	IM	160	35	HK/1/68	H3N2	10^7 EID50	35	640	21	Y	3	Nasal Wash	4.50	15	McLaren	1974	4522247
329		PR/8/34	H1N1	Infection		IN				35	HK/X31/68	H3N2	Vaccine	200 IU	IM	120	35	HK/1/68	H3N2	10^7 EID50	35	960	21	Y	3	Nasal Wash	2.83	15	McLaren	1974	4522247
331		PR/8/34	H1N1	Infection		IN				35	HK/X31/68	H3N2	Vaccine	200 IU	IM	60	35	HK/1/68	H3N2	10^7 EID50	35	1280	21	Y	3	Nasal Wash	3.50	15	McLaren	1974	4522247
333		PR/8/34	H1N1	Infection		IN				35	HK/X31/68	H3N2	Vaccine	200 IU	IM	120	35	HK/1/68	H3N2	10^7 EID50	35	480	21	Y	3	Nasal Wash	2.16	15	McLaren	1974	4522247
368		HK/X31/68	H3N2	Vaccine	200 IU	IM										<10	35	HK/1/68	H3N2	10^7 EID50	35	640	21	Y	3	Nasal Wash	5.50	15	McLaren	1974	4522247
370		HK/X31/68	H3N2	Vaccine	200 IU	IM										<10	35	HK/1/68	H3N2	10^7 EID50	35	2560	21	Y	3	Nasal Wash	5.50	15	McLaren	1974	4522247
371		HK/X31/68	H3N2	Vaccine	200 IU	IM										<10	35	HK/1/68	H3N2	10^7 EID50	35	960	21	Y	3	Nasal Wash	4.16	15	McLaren	1974	4522247
317		B/AA/66	B	Infection												<10	84	HK/1/68	H3N2	10^7 EID50	35	640	21	Y	3	Nasal Wash	5.50	15	McLaren	1974	4522247
318		B/AA/66	B	Infection												<10	84	HK/1/68	H3N2	10^7 EID50	35	64	21	Y	3	Nasal Wash	5.16	15	McLaren	1974	4522247
322		B/AA/66	B	Infection												<10	84	HK/1/68	H3N2	10^7 EID50	35	480	21	Y	3	Nasal Wash	5.50	15	McLaren	1974	4522247
325		B/AA/66	B	Infection												<10	84	HK/1/68	H3N2	10^7 EID50	35	960	21	Y	3	Nasal Wash	5.50	15	McLaren	1974	4522247
319		PR/8/34	H1N1	Infection												<10	84	HK/1/68	H3N2	10^7 EID50	35	960	21	Y	3	Nasal Wash	4.16	15	McLaren	1974	4522247
320		PR/8/34	H1N1	Infection												<10	84	HK/1/68	H3N2	10^7 EID50	35	1280	21	Y	3	Nasal Wash	4.50	15	McLaren	1974	4522247
321		PR/8/34	H1N1	Infection												<10	84	HK/1/68	H3N2	10^7 EID50	35	640	21	Y	3	Nasal Wash	4.83	15	McLaren	1974	4522247
323		PR/8/34	H1N1	Infection												<10	84	HK/1/68	H3N2	10^7 EID50	35	480	21	Y	3	Nasal Wash	4.16	15	McLaren	1974	4522247
352		NONE														<10	84	HK/1/68	H3N2	10^7 EID50	35	>5120	21	Y	3	Nasal Wash	5.16	15	McLaren	1974	4522247
359		NONE														<10	84	HK/1/68	H3N2	10^7 EID50	35	960	21	Y	3	Nasal Wash	4.50	15	McLaren	1974	4522247
363		NONE														<10	84	HK/1/68	H3N2	10^7 EID50	35	1920	21	Y	3	Nasal Wash	5.16	15	McLaren	1974	4522247
367		NONE														<10	84	HK/1/68	H3N2	10^7 EID50	35	960	21	Y	3	Nasal Wash	4.83	15	McLaren	1974	4522247
124		"HK vaccine"	H3N2	Vaccine	8000 HA units	IM										<5	35	HK/3/68	H3N2	10^6.5 EID50	35	320	28	Y	3	Nasal Wash	5.50	13	Potter	1972	4338059
127		"HK vaccine"	H3N2	Vaccine	8000 HA units	IM										<5	35	HK/3/68	H3N2	10^6.5 EID50	35	240	28	Y	3	Nasal Wash	5.00	13	Potter	1972	4338059
128		"HK vaccine"	H3N2	Vaccine	8000 HA units	IM										<5	35	HK/3/68	H3N2	10^6.5 EID50	35	640	28	Y	3	Nasal Wash	5.25	13	Potter	1972	4338059
114		"HK vaccine"	H3N2	Vaccine	8000 HA units	IM				14	"HK vaccine"	H3N2	Vaccine	8000 HA units	IM	<5	21	HK/3/68	H3N2	10^6.5 EID50	21	640	21	Y	3	Nasal Wash	4.75	13	Potter	1972	4338059
118		"HK vaccine"	H3N2	Vaccine	8000 HA units	IM				14	"HK vaccine"	H3N2	Vaccine	8000 HA units	IM	<5	21	HK/3/68	H3N2	10^6.5 EID50	21	480	21	Y	3	Nasal Wash	5.50	13	Potter	1972	4338059
121		"HK vaccine"	H3N2	Vaccine	8000 HA units	IM				14	"HK vaccine"	H3N2	Vaccine	8000 HA units	IM	<5	21	HK/3/68	H3N2	10^6.5 EID50	21	320	21	Y	3	Nasal Wash	4.50	13	Potter	1972	4338059
122		"HK vaccine"	H3N2	Vaccine	8000 HA units	IM				14	"HK vaccine"	H3N2	Vaccine	8000 HA units	IM	<5	21	HK/3/68	H3N2	10^6.5 EID50	21	320	21	Y	3	Nasal Wash	5.25	13	Potter	1972	4338059
119		"HK vaccine"	H3N2	Vaccine+ Adj. [Bord. Pert.Vacc]	8000 HA units	IM										80	28	HK/3/68	H3N2	10^6.5 EID50	35	1280	28	Y	3	Nasal Wash	3.50	13	Potter	1972	4338059
120		"HK vaccine"	H3N2	Vaccine+ Adj. [Bord. Pert.Vacc]	8000 HA units	IM										60	28	HK/3/68	H3N2	10^6.5 EID50	35	1280	28	Y	3	Nasal Wash	2.50	13	Potter	1972	4338059
125		"HK vaccine"	H3N2	Vaccine+ Adj. [Bord. Pert.Vacc]	8000 HA units	IM										15	28	HK/3/68	H3N2	10^6.5 EID50	35	960	28	Y	3	Nasal Wash	4.75	13	Potter	1972	4338059
109		"HK vaccine"	H3N2	Vaccine+Adj.[Freund's]	8000 HA units	IM										1280	35	HK/3/68	H3N2	10^6.5 EID50	35	2560	28	N	3	Nasal Wash	1.50	13	Potter	1972	4338059
117		"HK vaccine"	H3N2	Vaccine+Adj.[Freund's]	8000 HA units	IM										640	35	HK/3/68	H3N2	10^6.5 EID50	35	2560	28	Y	3	Nasal Wash	3.50	13	Potter	1972	4338059
113		"HK vaccine"	H3N2	Vaccine+Adj.[Freund's]	8000 HA units	IM										480	35	HK/3/68	H3N2	10^6.5 EID50	35	1920	28	Y	3	Nasal Wash	3.50	13	Potter	1972	4338059
116		"HK vaccine"	H3N2	Vaccine+Adj.[Freund's]	8000 HA units	IM										640	35	HK/3/68	H3N2	10^6.5 EID50	35	5120	28	Y	3	Nasal Wash	0.00	13	Potter	1972	4338059
253		HK/1/68	H3N2	Infection	10^6.5 EID50	IN										<5	35	PR/8/34	H1N1	10^6.5 EID50	35	>5120	21	Y				14	McLaren	1973	4769893
258		HK/1/68	H3N2	Infection	10^6.5 EID50	IN										<5	35	PR/8/34	H1N1	10^6.5 EID50	35	2560	21	Y				14	McLaren	1973	4769893
260		HK/1/68	H3N2	Infection	10^6.5 EID50	IN										<5	35	PR/8/34	H1N1	10^6.5 EID50	35	480	21	Y				14	McLaren	1973	4769893
261		HK/1/68	H3N2	Infection	10^6.5 EID50	IN										<5	35	PR/8/34	H1N1	10^6.5 EID50	35	2560	21	Y				14	McLaren	1973	4769893
285		PR/8/34	H1N1	Infection	10^6.5 EID50	IN										<5	35	HK/1/68	H3N2	10^6.5 EID50	35	30	6	Y				14	McLaren	1973	4769893
286		PR/8/34	H1N1	Infection	10^6.5 EID50	IN										<5	35	HK/1/68	H3N2	10^6.5 EID50	35	>5120	21	Y				14	McLaren	1973	4769893
287		PR/8/34	H1N1	Infection	10^6.5 EID50	IN										<5	35	HK/1/68	H3N2	10^6.5 EID50	35	>5120	21	Y				14	McLaren	1973	4769893
254		Aichi/2/68	H3N2	Vaccine	6400 CCA	IM										<5	35	HK/1/68	H3N2	10^6.5 EID50	35	5120	29	Y				14	McLaren	1973	4769893
255		Aichi/2/68	H3N2	Vaccine	6400 CCA	IM										<5	35	HK/1/68	H3N2	10^6.5 EID50	35	3840	29	Y				14	McLaren	1973	4769893
256		Aichi/2/68	H3N2	Vaccine	6400 CCA	IM										<5	35	HK/1/68	H3N2	10^6.5 EID50	35	3840	29	Y				14	McLaren	1973	4769893

258		Aichi/2/68	H3N2	Vaccine	6400 CCA	IM										<5	35	HK/1/68	H3N2	10^6.5 EID50	35	1280	29	Y				14	McLaren	1973	4769893
260		NONE														<5	35	HK/1/68	H3N2	10^6.5 EID50	35	320	29	Y				14	McLaren	1973	4769893
261		NONE														<5	35	HK/1/68	H3N2	10^6.5 EID50	35	960	29	Y				14	McLaren	1973	4769893

Notes:

Virus:

64b: PR/8/34 virus (H1N1) virus with the NA from Eng/939/69 (H3N2)

31a: PR/8/34 virus (H1N1) virus with the HA from Eng/939/69 (H3N2)

"HK Vaccine": a vaccine made described as 'formalin-killed, monotypic influenza virus A2/Hong Kong' in the paper (i.e. a 1968 Hong Kong H3N2 virus)

Treatment Dose (as reported by studies):

EID50: 50% Egg Infectious Dose

CCA: Chick Agglutinating Units

IU: Infectious Units

Treatment Route:

IM = Intramuscular

IN = Intranasal

Treatment Type:

With vaccines, one of two types of adjuvant was sometimes given: either Freund's or Bordetella Pertussis Vaccine

Ferrets:

The given reference numbers are supplied in the individual studies.

NB For ferrets from study [13], it is not totally clear how many days after challenge the HI measurements were taken. The methods state that it was as in [17] where they were done 28 days after challenge but the paper later explicitly states that this was done 3 weeks [i.e. 21 days] post challenge for ferrets 114, 118, 121 and 122. Of course, the timing of the HA measurements is not incorporated into this study, but is provided here for completeness.
